# Supplementary material for: Ginsenoside Rg3 Prevents Oncogenic Long Noncoding RNA ATXN8OS from Inhibiting Tumor-Suppressive microRNA-424-5p in Breast Cancer Cells
Source: Biomolecules. 2021 Jan 18;11(1):118. doi: 10.3390/biom11010118 (PMC7831931; doi:10.3390/biom11010118)
Supplement: Supplementary file 1 [file biomolecules-11-00118-s001.pdf]

Ginsenoside Rg3 prevents oncogenic long noncoding RNA ATXN8OS from inhibiting tumor-suppressive microRNA-424-5p in breast cancer cells

Heejoo Kim, Hwee Won Ji, Hyeon Woo Kim, Sung Hwan Yun, Jae Eun Park, and Sun Jung Kim

Supporting Information

Table S1. PCR primers, siRNA, miR-mimic, and miR-inhibitor used in this study.

Gene		Sequence (5′- 3′)	Supplier
**qRT-PCR**			
GAPDH	F :	ACATCGCTCAGACACCATG	IDT
	R :	TGTAGTTGAGGTCAATGAAGGG	
ATXN8OS	F :	GATTCTGGAGGCTGGGAAGT	Bionics
	R :	TCTTGCCCTTCTGCCTTCTA	
DACH1	F :	TGAACAAGCAGAACAGACGC	
	R :	ATCTGTTCTGCCGCCACT	
CHRM3	F :	GTGGCACCTGGTCTCTTTCT	
	R :	AGACCAGCTCCACCTGTT C	
U6		Hs_RNU6-2_11 miScript Primer Assay (MS00033740)	
has-miR-424-5p		Hs_miR-424_11 miScript Primer Assay (MS00004186)	
**Methylation Specific PCR**			
ATXN8OS	Methyl F :	ATG TTT GGA AGT TTC GTG GG	Bionics
	Unmethyl F :	ATG TTT GGA AGT TTT GTG GG	
	R :	ACTAATTCTACAAAAACTCAA A	
**siRNA**			

ATXN8OS #1	Sense :	CUCACAGAGUUUGAGUGUAtt	Bioneer
	Antisense :	UACACUCAAACUCUGUGAGtt	
ATXN8OS #2	Sense :	CUGAACACACAUAGUAGAAtt	
	Antisense :	UUCUACUAUGUGUGUUCAGtt	
control		Negative control siRNA (SN-1003)	
**miRNA mimic**			
has-miR-424-5p	Sequence :	CAGCAGCAAUUCAUGAA	Bioneer
control		miRNA Negative control, mimic #1 (SMC-2002)	
**miRNA inhibitor**			
has-miR-424-5p	Sequence :	CAGCAGCAAUUVAUGUUUUGAA	Bioneer
control		miRNA Negative control, inhibitor #1 (SMC-3102)	

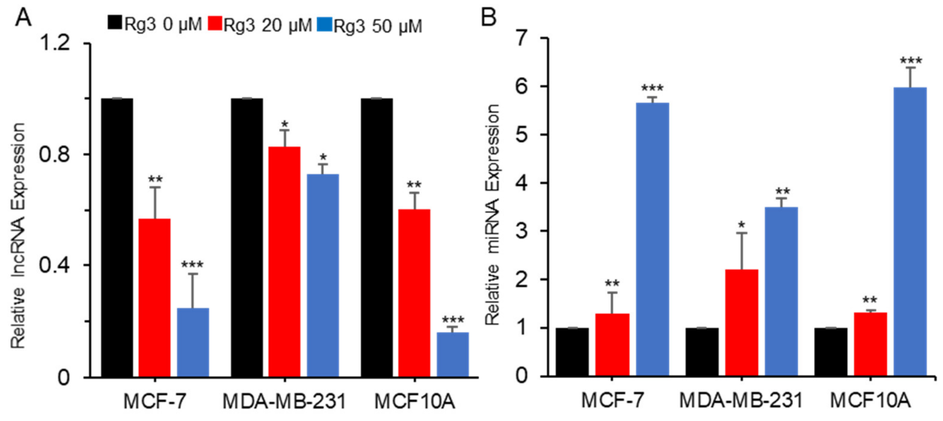

Figure S1. Regulation of ATXN8OS and miR-424-5p by Rg3 in mammary gland-derived cell lines. Breast cancer cell lines, MCF-7 (ER-positive) and MDA-MB-231 (ER-negative), and a normal breast cell line (ER-positive) were treated with Rg3, and the expression of ATXN8OS (A) and miR-424-5p (B) was examined by qRT-PCR. All experiments were performed in triplicate, and the values are presented as the mean ± SE. *P < 0.05, **P < 0.01, ***P < 0.001

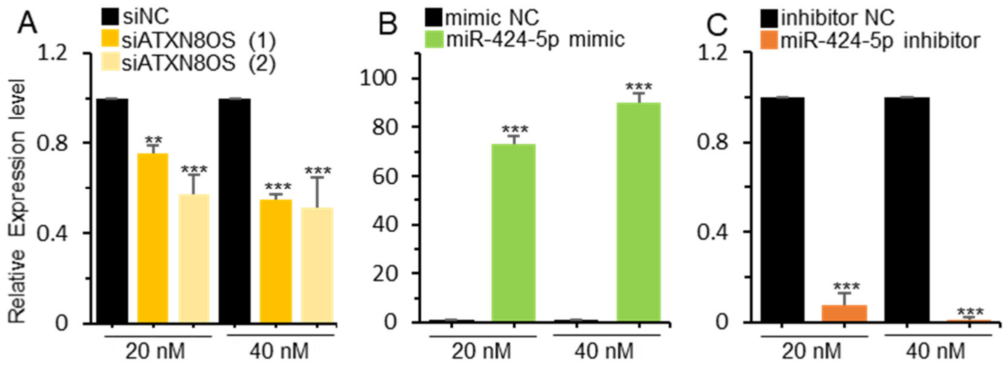

Figure S2. Induction of deregulation of ATXN8OS and miR-424-5p in MCF-7. Cultured cells were transiently transfected with one of the two siRNAs targeting different sites of ATXN8OS (A), miR-424-5p mimic (B), and miR-424-5p inhibitor (C). Gene expression was examined by qRT-PCR. siNC, control siRNA; mimic NC, negative control mimic for miR-424-5p; inhibitor NC, negative control inhibitor for miR-424-5p. All results are reported as the means ± SE of three experiments. **P < 0.01, ***P < 0.001

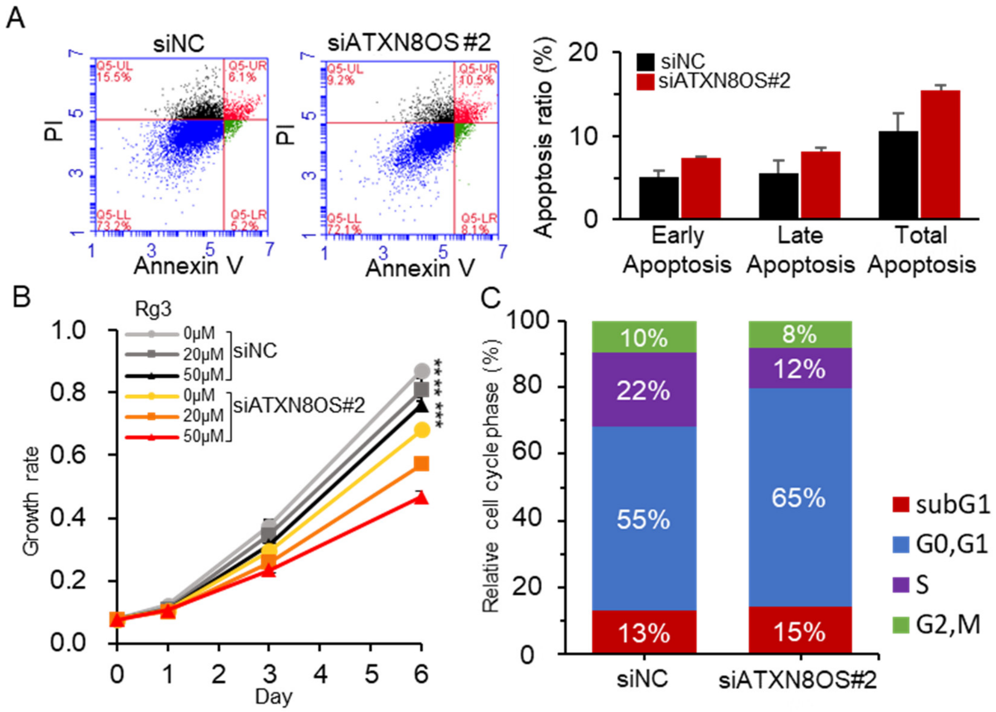

Figure S3. Effect of ATXN8OS on apoptosis, cell growth, and cell cycle. An siRNA of ATXN8OS (siATXN8OS#2) targeting different site from the one (siATXN8OS#1) used in the main manuscript was transiently transfected to MCF-7 cells and its effect on apoptosis (A), cell growth (B), and cell cycle (C) was monitored. Experiments were performed in triplicate, and the values are presented as the mean ± SE. siNC, control siRNA. **P < 0.01, ***P < 0.001

A

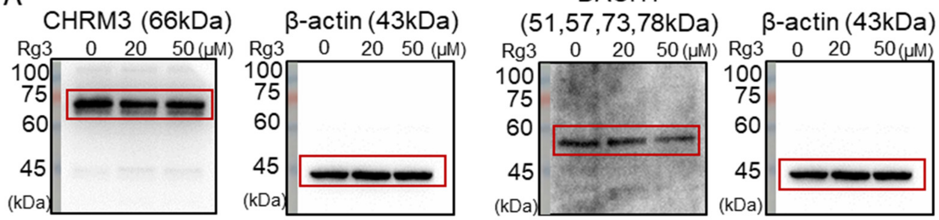

Rg3	0 μM	20 μM	50 μM
β-actin	6,031,619	7,244,017	7,619,593
CHRM3	12,547,952	10,601,092	10,754,138

Rg3	0 μM	20 μM	50 μM
β-actin	6,031,619	7,244,017	7,619,593
DACH1	1,957,795	1,696,826	1,851,350

B

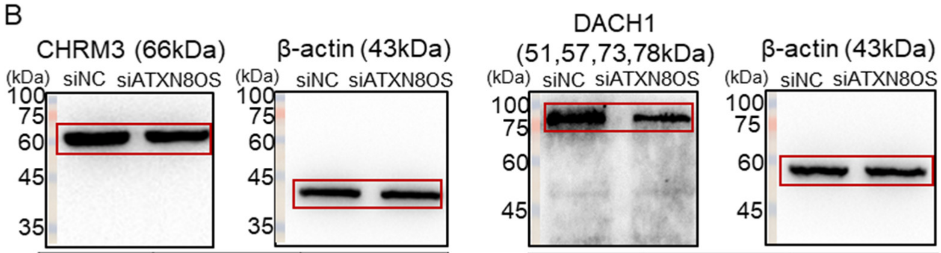

	siNC	siATXN8OS
β-actin	6,892,352	6,548,780
CHRM3	5,892,860	3,784,548

	siNC	siATXN8OS
β-actin	14,967,564	14,255,768
DACH1	13,640,180	7,838,180

C

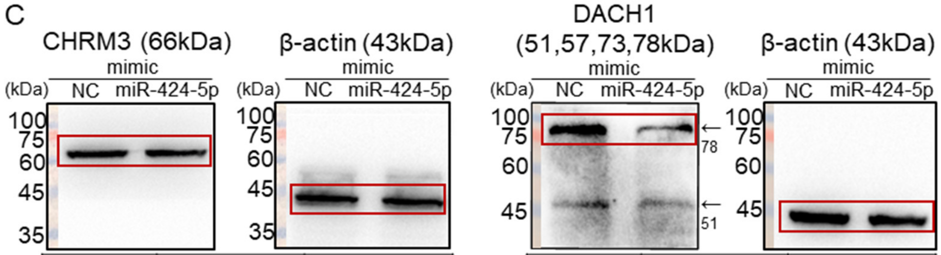

	mimic NC	miR-424-5p mimic
β-actin	6,892,352	6,548,780
CHRM3	5,892,860	3,784,548

	mimic NC	miR-424-5p mimic
β-actin	10,293,531	7,070,843
DACH1	2,865,092	752,628

D

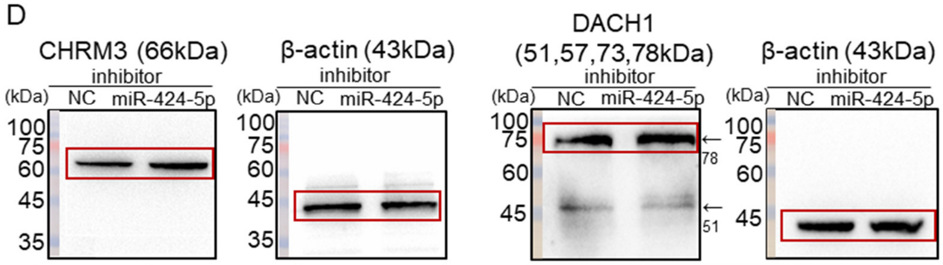

	inhibitor NC	miR-424-5p inhibitor
β-actin	16,367,320	15,250,760
CHRM3	14,976,708	17,897,224

	inhibitor NC	miR-424-5p inhibitor
β-actin	8,801,060	8,275,068
DACH1	4,893,529	5,994,351

Figure S4. Uncropped Western blots. The boxed areas correspond to the images in the main text. Protein is extracted after Rg3 treated (A), transiently transfected with a siRNA to ATXN8OS (B), miR-424-5p mimic (C), and miR-424-5p inhibitor (D).
